# Mode of Application of Peracetic Acid-Based Disinfectants has a Minimal Influence on the Antioxidant Defences and Mucosal Structures of Atlantic Salmon (*Salmo salar*) Parr

**DOI:** 10.3389/fphys.2022.900593

**Published:** 2022-05-25

**Authors:** Danilo Carletto, Francisco Furtado, Junjie Zhang, Alexandros G. Asimakopoulos, Maia Eggen, Gerhardus C. Verstege, Caterina Faggio, Vasco C. Mota, Carlo C. Lazado

**Affiliations:** ^1^ Nofima AS, Norwegian Institute of Food, Fisheries and Aquaculture Research, Tromsø, Norway; ^2^ Department of Chemical, Biological, Pharmaceutical and Environmental Sciences, University of Messina, Messina, Italy; ^3^ CIISA, Faculty of Veterinary Medicine, University of Lisbon, Lisbon, Portugal; ^4^ Department of Chemistry, The Norwegian University of Science and Technology (NTNU), Trondheim, Norway; ^5^ Faculty of Biosciences, Fisheries and Economics, UiT The Arctic University of Norway, Tromsø, Norway; ^6^ Havbruksstasjonen i Tromsø AS, Kårvik, Norway; ^7^ Nofima AS, The Norwegian Institute of Food, Fisheries and Aquaculture Research, Tromsø, Norway

**Keywords:** atlantic salmon, fish health, mucosal immunity, oxidative stress, peracetic acid disinfection, recirculating aquaculture systems (RAS)

## Abstract

Peracetic acid (PAA) is an oxidative disinfectant with a broad spectrum of antimicrobial activity and low environmental impact. In this study, we investigated the physiological impacts of PAA application in Atlantic salmon (*Salmo salar*) parr reared in freshwater recirculating aquaculture systems over a 4-week period. PAA at a target concentration of 1 mg/L was administered either in pulse (every 3 days) or continuous. The group that did not receive PAA served as a control. Fish tissue samples were collected for histology, gene expression, and biochemical analyses at day 0 and after 2 and 4 weeks of exposure. The expression of genes encoding for antioxidant defence in the olfactory organs, skin, and gills changed during the trial, but the temporal effects were more pronounced than inter-treatment impacts. The glutathione group of antioxidant genes was more responsive to PAA. In most cases, an upregulation was observed. Significantly lower levels of reactive oxygen species were identified in the plasma and skin mucus of the two PAA-exposed groups at week 4; nonetheless, significantly increased levels of total antioxidant capacity were only observed in the skin mucus of fish from the continuous treatment group. Additional markers of oxidative stress (i.e., 8-oxo-2′-deoxyguanosine and o,o'-dityrosine) were analysed in the skin, gills, liver, and dorsal fins. These markers were unaffected by the two PAA treatments. Sporadic reversible structural alterations were observed in the three mucosal organs; the changes were time-dependent, and the effects of PAA treatment were minimal. The number of mucous cells varied over time but not within treatments except in the skin of the pulse group at week 4 where a reduction was observed. The ratio of acidic and neutral mucous cells in the skin and gills were affected by PAA treatments especially in the pulse group. Overall, this study revealed that Atlantic salmon parr mobilised mucosal and systemic antioxidant defences against the oxidative disinfectant PAA, but it was evident that the mode of application did not impose a strong influence. The minimal effects of PAA application on the indicators of health and welfare underscore the potential use of PAA as a routine disinfectant in recirculating aquaculture systems.

## 1 Introduction

Norwegian Atlantic salmon aquaculture has developed and expanded significantly in the last 2 decades, thus making Norway a top producer with a share of at least 50% of the global salmon production (FAO, 2020). The success of this enormous expansion is mainly attributed to the high market value of salmon combined with technological innovations that enable higher production with efficient yields. A major technological innovation in recent years is the adoption of recirculating aquaculture systems (RAS) that facilitate efficient control of the rearing environment, i.e., these systems have high biosecurity control.

Ensuring optimal water quality to support the biological requirements of fish is fundamental to the successful implementation of RAS-based farming (Lazado et al., 2021a). RAS is generally an intensive production, i.e., high feeding rates. This leads to high organic loads and the accumulation of particles (Mota et al., 2015). The RAS environment is a closed system with reused water containing a high organic load; thus, it is a hotspot for opportunistic pathogens that could prompt disease outbreaks in cases where conditions are sub-optimal (Rurangwa and Verdegem, 2015). Pathogen challenges can still be an issue in RAS because they can derive from various sources including make-up water, feed, fish stock, dirty equipment, etc. (Blancheton et al., 2013; De Schryver and Vadstein, 2014). Therefore, routine water disinfection is an integral system component to address this issue.

Disinfectants that offer a broad spectrum of antimicrobial activity, minor environmental risk, and negligible impact to fish health and welfare are ideal. Peracetic acid (PAA) has recently gained prominence and is an eco-friendly disinfectant in aquaculture. PAA is a potent organic peroxide and is available commercially as an acidified mixture of acetate and hydrogen peroxide. Its antimicrobial activity is mainly attributed to the denaturation of protein, disruption of cell wall permeability, and oxidation of sulfhydryl and sulphur bonds in proteins, enzymes, and other metabolites. Some of the key characteristics of PAA that makes it highly favourable for aquaculture use include rapid action against a wide array of pathogens, lack of harmful decomposition products (i.e., acetic acid, water, oxygen, hydrogen peroxide), inert residues, effectiveness in the presence of organic matter, sporicidal even at low temperatures, and low pH dependence (Jussila et al., 2011; Domínguez Henao et al., 2018; Ao et al., 2021). These features make PAA advantageous compared to other major disinfectants such as chlorine, which generate toxic, mutagenic, and carcinogenic by-products (Ding et al., 2013; Rodrigues Macêdo et al., 2019).

PAA is an oxidising agent and produces free radicals upon contact with organic compounds. Though this innate feature is attributed to its disinfection power, higher levels of radicals in the water exert a physiological pressure on the fish (Soleng et al., 2019). Reactive oxygen species (ROS) are highly active forms of oxygen molecule with a dual biological function: They normally are produced as a by-product of metabolism and function as signalling molecules, thus regulating various cellular processes (Schieber and Chandel, 2014). The exogenously-generated form may trigger oxidative stress and pose a health risk when not managed properly. Oxidative stress occurs when the ROS concentration surpasses the capacity of antioxidants to sequester and neutralise these reactive molecules. It may cause various types of damage to cells, proteins, and DNA. In serious cases, it can lead to disease. PAA can induce oxidative stress in fish as documented by altering the internal ROS balance and/or affecting the activity of antioxidant molecules both at transcript and protein levels (Liu et al., 2020; Osório et al., 2022). Therapeutic doses of PAA have been documented to induce transient oxidative stress particularly in salmon smolts. The mobilisation of antioxidants both at the systemic and mucosal levels could be both spontaneous and latent depending on the dose and mode of delivery. We previously reported that the application of PAA in brackish water salmon RAS could be a mild environmental stressor; nonetheless, the fish could mount strong adaptive responses to routine application (Osório et al., 2022).

Here, we investigated the health and welfare consequences of PAA in Atlantic salmon parr reared in a RAS. There is substantial evidence regarding the physiological consequences of PAA in salmon post-smolts but not during the parr stage. The efficacy of PAA is affected by different factors including life stage, dose, water quality, etc.; therefore, extrapolating the health consequences in post-smolts to parr is not ideal. A recent study reported that the no-effect concentration of PAA for Atlantic salmon parr is below 1.6 mg/L (Mota et al., 2022a). Using two administration methods, i.e., pulse or continuous, we focused on the physiological cost of the PAA application towards how salmon mount antioxidant responses to the presence of radicals in the water and the tissue structural alterations following its use. We employed molecular, biochemical, and histological approaches to profile the response to salmon parr to PAA.

Environmental stressors impact fish mucosa—mucosa is a key structure in the host-environment interaction and is often considered the first line of defence in fish. Several studies have reported that the salmon mucosa is highly vulnerable to exogenous radicals such as PAA. The mucosal organs of salmon are highly sensitive to PAA even at very low concentrations (e.g., 0.6 mg/L) (Lazado et al., 2020a; Mota et al., 2022a); hence, this work focused on response profiling on gills, skin, and olfactory organs.

## 2 Materials and Methods

### 2.1 Ethical Use of Animals in Research

The study adhered to the guidelines and protocols of the European Union Directive 2010/63/EU and the National Guidelines for Animal Care and Welfare established by the Norwegian Ministry of Education and Research. The experiment was approved by the Norwegian Food Safety Authority under FOTS ID 24128. The key personnel handling the fish hold a FELASA C certificate.

### 2.2 Experimental Fish

The experiment was conducted at Tromsø Aquaculture Research Station (HiT, Kårvik, Norway). Atlantic salmon (*Salmo salar*) were hatched and reared until the appropriate experimental size using the standard procedures at HiT. Briefly, Atlantic salmon eyed eggs were hatched and raised in a freshwater flow-through system at 10°C, oxygen saturation >85%, and a photoperiod regime of continuous illumination (LD 24:00) until transfer to the experimental RAS units. Fish were fed to apparent satiation with a commercial diet for Atlantic salmon parr (1 mm pellet size, Nutra Olympic, Skretting, Norway).

### 2.3 Experimental Set-Up

Technical information about the RAS units is provided in detail in Mota et al., 2022b; Mota et al., 2022b. Briefly, each experimental unit consisted of a cylindro-conical fish tank (V = 0.5 m^3^) connected to a water treatment system composed of a drum filter (micro-screen mesh size = 40 µm), a moving bed bioreactor (V = 0.2 m^3^, 750 m^2^/m^3^ bio-media), a gas exchange unit (CO_2_-degasser cylinder), a foam fractionator, low pressure oxygen cone (0.6 Bar), and a temperature control unit. The total RAS water volume was 0.8 m^3^. The water flow to the fish tank was 1500 L/h, and the fish tank hydraulic retention time was 20 min. Continuous illumination was provided by a LED light above each unit.

The trial employed nine individual RAS units, and each tank unit was stocked with 40 fish (start weight 24.7 ± 3.4 g). Fish were allowed to acclimatise for 2 weeks under the following environmental conditions, which were also maintained throughout the entire experiment: dissolved oxygen >85% saturation, pH 7 - 7.5, temperature = 11.5 - 12.5°C, salinity = 2 ppt. Fish were fed continuously with a commercial diet (1.5 mm pellet size, Nutra Olympic, Skretting, Norway) delivered through an automatic belt feeder.

There were three experimental groups: a control and two experimental groups that received 1.0 mg/L peracetic acid (PAA, 6.6% v/v PAA, 23.4% hydrogen peroxide, and 10% acetic acid, Aqua Des ™, Aquatic Chemistry AS, Lillehammer, Norway) either in pulse or continuous mode. The actual PAA concentration in the product was empirically verified by an external laboratory (DTU Aqua, through Dr. Lars-Flemming Pedersen). Each group had three replicate RAS units. PAA was administered directly into the pump sump of the two PAA-exposed groups via a high precision multichannel peristaltic pump (IPC, ISMATEC®, Cole-Parmer, United States). PAA was provided every 3 days in the pulse group; the same amount of PAA was given to the continuous group every 3 h over a 24 h period. The PAA exposure trial lasted for 4 weeks. The average fish weight at termination was 53.9 ± 6.6 g.

### 2.4 Tissue Sampling

There were three extensive sampling points to assess the effects of PAA exposure treatment on fish: before the start of the PAA addition (week 0), mid-way of the trial (week 2), and at termination (week 4). Three fish were taken at week 0 as well as five in the next two sampling points (2 and 4 weeks) from each individual tank. Fish were humanely euthanised by bathing in an overdose of benzocaine (Benzoak vet, 200 mg/ml, EuroPharma, Norway). The fork length and weight were measured. Skin mucus was collected by a FLOQSwabs® swab (COPAN Diagnostics, United States) below the lateral line, snap frozen in dry ice, and then eventually stored at −80°C. Blood was withdrawn from caudal artery by a heparinised vacutainer (BD Vacutainer™, United States) and centrifuged for 10 min at 2,000 rpm; plasma was collected and stored at −80°C.

Sections of dorsal skin (just below the dorsal fin), the second gill arch, and the olfactory organ were dissected and divided into two portions. The first fraction was suspended in RNAlater™ (Ambion, United States), kept at room temperature overnight to allow penetration, and then stored at −80°C until RNA extraction. The other half of the dissected dorsal skin, olfactory organ, and second gill arch was stored in 10% neutral buffered formalin (BiopSafe®, Denmark). In addition to gills and skin, liver and dorsal fin were also collected, snap frozen in dry ice, and stored at −80°C.

### 2.5 RNA Isolation, Quantification and Quality Control

The total RNA was isolated from the gills, skin, and olfactory organ from all three time points (week 0, 2, and 4) in Biomek 4,000 Benchtop Workstation using Agencourt RNAdvance™ Tissue Total RNA Purification Kit (Beckman Coulter Inc, United States). The concentration and purity were determined through NanoDrop 8,000 spectrophotometer (Thermo Scientific, United States). The quality of a representative number of samples was further verified using an Agilent^®^ 2,100 Bioanalyzer™ RNA 6000 Nano kit (Agilent Technology Inc, United States). All assessed samples had an RNA Integrity Number (RIN) above 8.0.

### 2.6 Reverse Transcription (RT) and cDNA Synthesis

The cDNA synthesis used a TaqMan™ Reverse Transcription Kit (Applied Biosystems, United States) using a 500-ng input RNA in a 20 μL reaction. The synthesis was performed using a Veriti™ 96-Well Thermal Cycler 7 (Applied Biosystems, United States) with the following parameters: 10 min at 25°C, 30 min at 37°C, and 5 min at 95°C.

### 2.7 Real-Time Quantitative Polymerase Chain Reaction (RT-qPCR)

QuantStudio™ Real-Time PCR System (Applied Biosystems, United States) was used to perform RT-qPCR. The 10 μL reaction mixture contained the following: 5 μL of PowerUp™ SYBR™ Green Master Mix (Applied Biosystems, United States) and 0.5 μL 10 μM of each forward/reverse primer (Invitrogen, United States) as well as 4 μL 1:10 diluted cDNA. All samples were run in duplicate. The thermocycling parameters were as follows: 20 s of pre-incubation at 95°C, amplification with 40 cycles at 95°C for 1 s and 60°C for 20 s. A dissociation stage for 1 s followed at 95°C, 20 s at 60°C, and 1 s at 95°C. A five-step standard curve of 2-fold dilution series was prepared from pooled cDNA to determine the amplification efficiencies. The expression of six target genes including *catalase* (*cat*), *glutathione S-transferase* (*gsta*), *glutathione reductase* (*gr*), *glutathione peroxidase* (*gpx*), *copper/zinc superoxide dismutase* (*zn/cu sod*), and *manganese superoxide dismutase* (*mnsod*) were normalised against two housekeeping genes including *elongation factor alpha-1 (ef1α)* and *β-actin* (*actb*) as reported earlier (Nagasawa et al., 2012). All primer sequences are provided in Table 1.

**TABLE 1:** List of the gene primers used in this study.

Gene Name	Abbreviation	Sequences (5’→3′)	References
*Elongation factor alpha 1*	*ef1a*	F: GAA​TCG​GCT​ATG​CCT​GGT​GAC	Garcia De La Serrana and Johnston (2013)
		R: GGA​TGA​TGA​CCT​GAG​CGG​TG	
*B-actin*	*actb*	F: CCA​AAG​CCA​ACA​GGG​AGA​A	Sanden and Olsvik (2009)
		R: AGG​GAC​AAC​ACT​GCC​TGG​AT	
*Glutathione S-transferase*	*gsta*	F: AGG​GCA​CAA​GTC​TAA​AGA​AGT​C	Lazado and Voldvik (2020)
		R: GTC​TCC​GTG​TTT​GAA​AGC​AG	
*Copper/Zinc superoxide dismutase*	*cu/zn sod*	F: CCACGTCCATGCCTTTGG	Solberg et al. (2012)
		R: TCA​GCT​GCT​GCA​GTC​ACG​TT	
*Manganese superoxide dismutase*	*mn sod*	F: GTT​TCT​CTC​CAG​CCT​GCT​CTA​AG	Solberg et al. (2012)
		R: CCG​CTC​TCC​TTG​TCG​AAG​C	
*Catalase*	*cat*	F: GGG​CAA​CTG​GGA​CCT​TAC​TG	Olsvik et al. (2011)
		R: GCA​TGG​CGT​CCC​TGA​TAA​A	
*Glutathione reductase*	*gr*	F: CCA​GTG​ATG​GCT​TTT​TTG​AAC​TT	Solberg et al. (2012)
		R: CCGGCCCCCACTATGAC	
*Glutathione peroxidase*	*gpx*	F: GAT​TCG​TTC​CAA​ACT​TCC​TGC​TA	Solberg et al. (2012)
		R: GCT​CCC​AGA​ACA​GCC​TGT​TG	

### 2.8 Histological Evaluation of Mucosal Organs

The formalin-preserved gills and skin tissue samples were processed by an external laboratory (Norwegian Veterinary Institute, Harstad, Norway). These tissue sections were stained by Periodic Acid Schiff-Alcian Blue (AB-PAS) and delivered to Nofima as digital scanned files. The olfactory organ samples were processed in-house. The tissue samples were decalcified in 10% Titriplex (Sigma Aldrich), processed in an automated tissue processor (TP1020, Leica Biosystems, Germany), and embedded in paraffin (Leica EG1150H, Leica Biosystems, Nussloch, Germany). A 5-μm section was prepared for each sample in a rotatory microtome (Leica RM2165, Leica Biosystems, Nussloch, Germany). This was AB/PAS-stained using an automated stainer (ST5010, Leica Biosystems) (Osório et al., 2022). The stained slides were digitised using a slide scanner (Aperio CS2 slide scanner, Leica Biosystem).

The tissue sections were assessed using scoring schemes to identify the health status and integrity of the organs. The skin sections were evaluated using two general criteria: general appearance of the epidermis and quality of the epithelial surface. A semi-quantitative three-point skin health scoring system described in other previous work was used (Sveen et al., 2018; Stiller et al., 2020). For the gills, four regions in the section were randomly selected, and each region contained 40 lamellae thereby accounting for a total of 160 lamellae analysed per fish. Thereafter, cases of branchial pathologies including epithelial lifting, lamellar clubbing, hyperplasia, hypertrophy, lamellar fusion, necrosis, and aneurysm were accounted. Lamellae that did not show any of the lesions were defined as healthy (Lazado et al., 2021a). A scoring scheme was arbitrarily developed for the olfactory organ in this study (Supplementary Table S1). Briefly, the tissue sections were scored using a 0-to-3 scoring scheme where 0 means no apparent abnormalities or lesions, and three means severe damage including extensive loss of structure.

Mucous cells were further quantified in the skin and gills. Three regions of the skin were selected each of around 2,000 μm in distance. Epidermal mucous cells were quantified and differentiated as acidic or neutral. For the gills, mucous cells were accounted in the regions selected for pathological scoring as described above. Mucous cells were counted both in the lamella and filament and were further differentiated as either acidic or neutral.

A quantitative morphometric evaluation was performed on three randomly selected olfactory lamellae per fish. The thickness of the olfactory epithelium and lamina propria were measured in six locations for the epithelium, and three locations for the lamina propria. The width of the olfactory lamellar tip was also evaluated.

### 2.9 Determination of Total Antioxidant Capacity (TAC) in Plasma and Skin Mucus

The determination of TAC both in plasma and skin mucus was performed via a colorimetric kit (Sigma-Aldrich, United States). Only samples collected at week 4 were used in the analysis. This assay determines the concentration of small molecules and antioxidant proteins or the concentration of only small molecule antioxidants as validated earlier for salmon (Soleng et al., 2019). The level of antioxidant capacity is expressed relative to 6-hydroxy-2,5,7,8-tetramethylchroman-2-carboxylic acid (Trolox). Due to the low amount of skin mucus, the quantification used a pooled sample of five fish per replicate tank.

### 2.10 Determination of Reactive Oxygen Species (ROS) and Reactive Nitrogen Species (RNS) in Plasma and Skin Mucus

To determine the ROS and RNS concentration in plasma and skin mucus, OxiSelect™ *In Vitro* ROS/RNS Assay Kit was used (Cell Biolabs Inc.), United States . The kit was suitable to detect and measure the total reactive oxygen species (ROS) plus reactive nitrogen species (RNS) in a wide variety of sample types such as plasma and cell lysates. The level of ROS and RNS is determined by the oxidation level of the probe dichlorodihydrofluorescin DiOxyQ (DCFH-DiOxyQ) expressed in terms of fluorescence. Hydrogen peroxide (H_2_O_2_) was used as a standard.

### 2.11 Determination of the Oxidative Stress Biomarkers of 8-Hydroxydeoxyguanosine (8OHDG) and Dityrosine (DIY) in Gills, Skin, Dorsal Fin, and Liver Tissue

The tissue samples were extracted and analysed based on the procedure reported by Zhang et al. (2022) (Zhang et al., 2022). Briefly, samples were thawed in room temperature, and a ∼100-mg portion of each tissue sample was obtained. Next, 600 μL methanol containing 1% ammonium formate (w/v) was added, and the samples were vortex mixed for 30 s, ultrasonicated for 30 min, and centrifuged for 5 min at 3,500 rpm. The supernatant was collected for further sample purification: The supernatant (extract) was transferred for UPLC^®^-MS/MS analysis. UPLC^®^-MS/MS data was acquired with the MassLynx v4.1 software, and quantification processing was performed with TargetLynx (Waters, Milford, United States).

### 2.12 Data Handling and Treatment

The normal distribution was evaluated using a Shapiro-Wilk test, and then a Brown-Forsythe test analysed equal variance of the data from plasma, total antioxidant capacity analysis, reactive oxygen species analysis, gene expression analysis, and histological assessment (Systat Software Inc, London, UK). A two-way analysis of variance (ANOVA) was used to evaluate differences within modes of exposure. We further evaluated the sampling points and the interactions between these two factors including for gene expression and histology data. A one-way ANOVA was also used to test for differences among treatments for the plasma and mucus ROS/RNS and TAC analysis. In both cases, a Holm-Sidak method comparison was performed when significant differences were observed. Principal component analysis (PCA) was performed with Origin 2019 (OriginLab Corporation, Northampton, MA, United States 2019). Pearson correlation analysis was performed with SPSS (version 28.0.1.0.0). The statistical level of variance was set at *p* < 0.05. All data are presented as mean ± standard deviation (S.D.).

## 3 Results

### 3.1 The Expression of Key Antioxidant Defence Genes in the Gills

**FIGURE 1:**
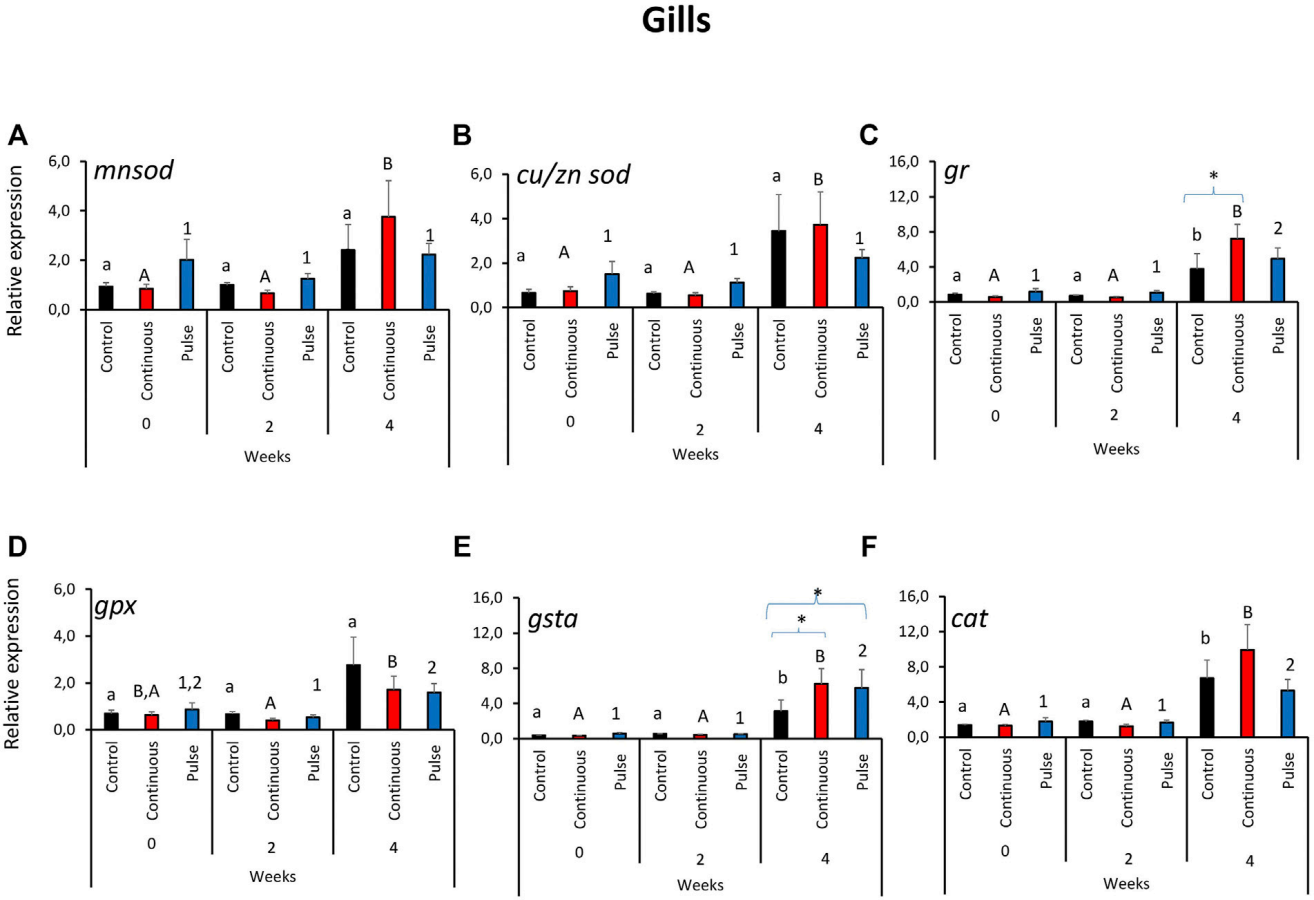
Relative expression of key antioxidant defence genes in the gills. **(A)** *manganese superoxide dismutase* (*mnsod*), **(B)** *copper/zinc superoxide dismutase* (*zn/cusod*), **(C)** *glutathione reductase* (*gr*), **(D)** *glutathione peroxidase* (*gpx*), and **(E)** *glutathione S-transferase* (*gsta*); **(F)** *catalase* (*cat*). Significant differences over time are represented by different lower-case letters (for control group), upper case letters (for continuous group), or numbers (for pulse group). Significant differences between treatment groups at a particular time are indicated by an asterisk (*). Values are presented as mean ± SD of nine individual fish per sampling point.

The expression of the antioxidant defence genes in the gills was predominantly affected by time and not by the treatments (Figure 1). The transcription of *mnsod* and *cu/zn sod* in the gills showed a similar tendency where the level in the continuous group was significantly higher at week 4 than the 2 earlier time points (Figures 1A, B). No significant inter-treatment differences were observed in the gill expression of *mnsod* and *cu/znsod*. The expression of *gpx* and *cat* was significantly elevated in both PAA-exposed groups at week 4 versus weeks 0 and 2; nonetheless, no significant inter-treatment difference was observed (Figures 1D, F). In addition, the *cat* transcript level in the control group at week 4 was significantly different from weeks 0 and 2 (Figure 1F). The expression of *gr* (Figure 1C) and *gsta* (Figure 1E) showed significant temporal changes where the transcript level in the PAA treatment groups, including the control, was significantly different from two earlier time points. Inter-treatment differences were further identified at week 4: The expression of *gr* in the continuous group was significantly different than the control, while the transcript levels of both *gr* and *gsta* were significantly higher in continuous and pulse groups than the control group.

### 3.2 The Expression of Key Antioxidant Defence Genes in the Skin

**FIGURE 2:**
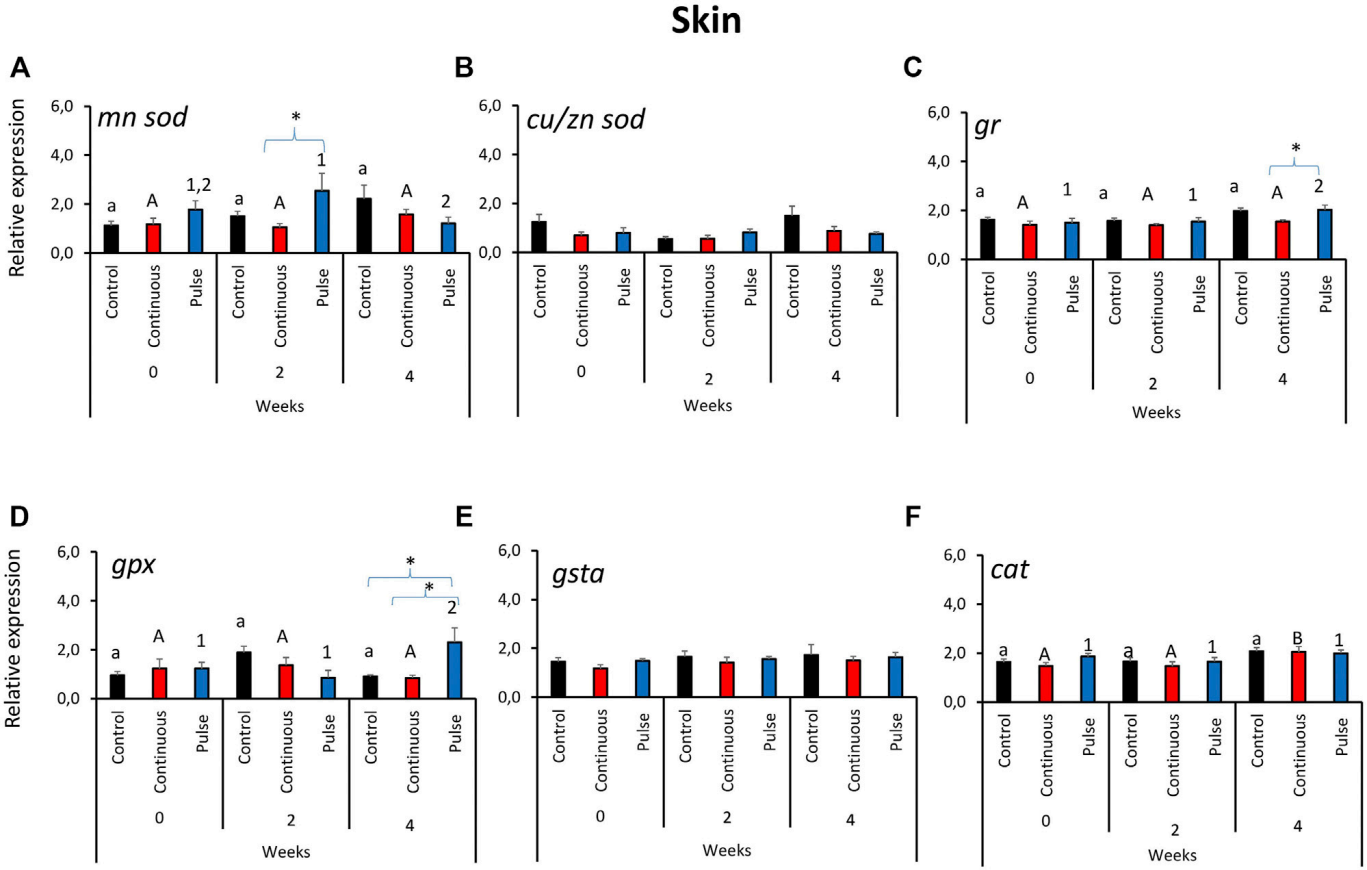
Relative expression of key antioxidant defence genes in the skin. Kindly refer to Figure 1 for information about statistical notations.

Unlike in the gills, the gene expression profile of antioxidant genes in the skin did not reveal a clear overall pattern (Figure 2). The expression of *mnsod* in the pulse group was significantly elevated at week 2 versus weeks 0 and 4 (Figure 2A). We further showed that the *mnsod* transcript level in the pulse group was significantly higher than the continuous group but not the control group at week 2. Significant temporal variation in *mnsod* expression was not observed in the control or continuous groups. The expression of *gr* (Figure 2C) and *gpx* (Figure 2D) was significantly higher in the pulse group at week 4 than the two earlier timepoints. Significant inter-treatment differences were observed at termination: The *gr* expression in the pulse group was significantly different from the continuous group (Figure 2C), while *gpx* expression in the pulse group was significantly different from the continuous and control groups. Though no inter-treatment differences were observed in *cat* expression, the transcription in the continuous group at termination was significantly different from the two previous timepoints (Figure 2F). Neither *cu/znsod* (Figure 2B) nor *gsta* (Figure 2E) had significant temporal and inter-treatment differences.

### 3.3 The Expression of Key Antioxidant Defence Genes in the Olfactory Organ

**FIGURE 3:**
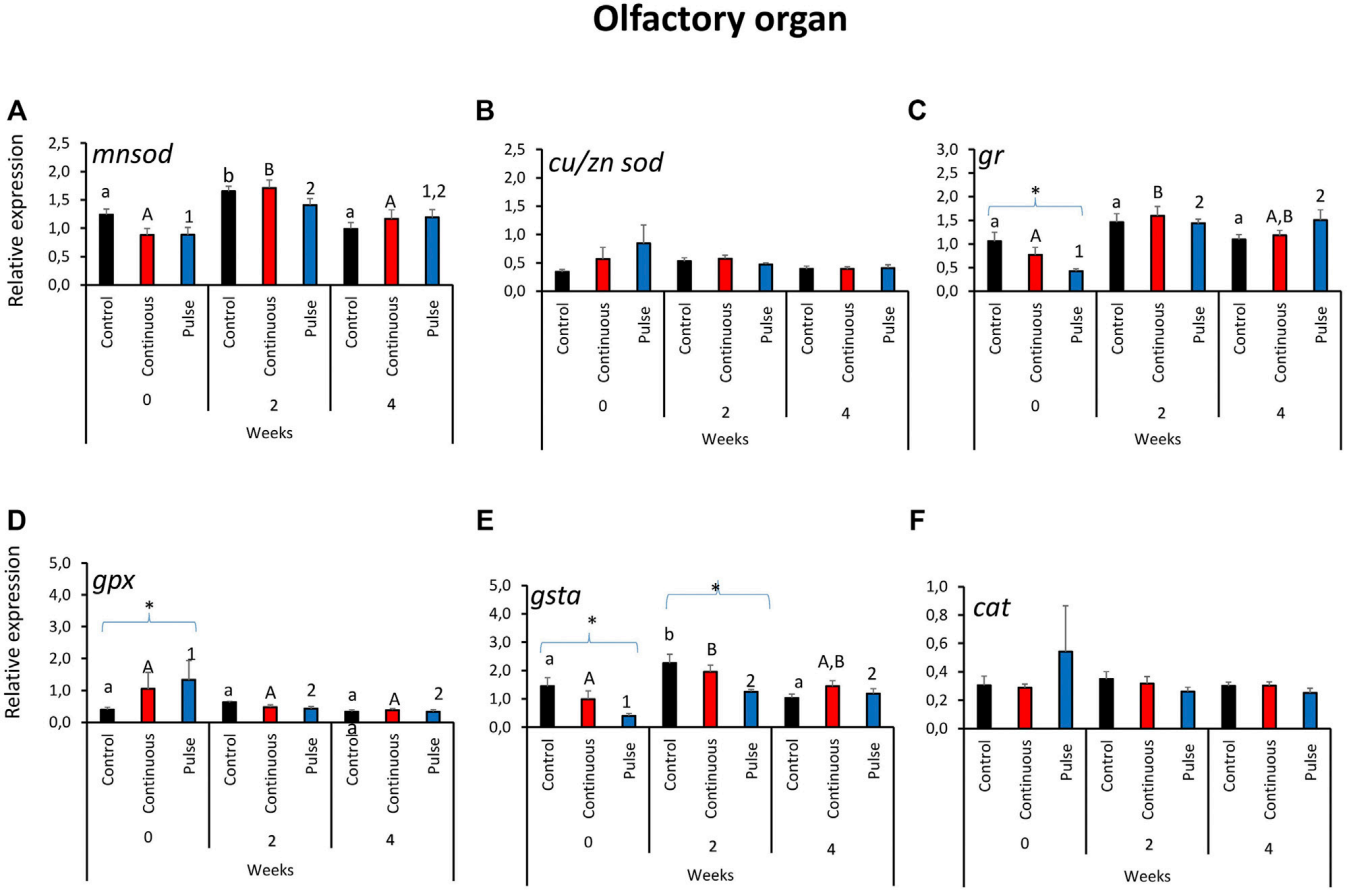
Relative expression of key antioxidant defence genes in the olfactory organ. Kindly refer to Figure 1 for information about statistical notations.

Significant temporal variations were found in the expression of *mnsod* in all treatment groups (Figure 3A). The expression of *mnsod* in both the control and continuous groups were significantly elevated at week 2 compared with the two other timepoints. The transcript level of *mnsod* in the pulse group was also significantly higher at week 0 than week 2 but not week 4. The expression of *gr* demonstrated temporal and inter-treatment variations (Figure 3C); expression in the continuous group was significantly higher at week 2 than week 0.

In the pulse group, elevated expression was observed in both weeks 2 and 4 versus week 0. At the start of the exposure trial, the expression of *gr* in the pulse group was significantly lower than the control. Significant inter-treatment differences were also observed in *gpx* expression at the start; the pulse group was significantly higher than the control (Figure 3D). Moreover, *gpx* expression at week 0 was significantly higher than the levels observed in the two subsequent timepoints. The *gsta* expression showed both temporal and inter-treatment differences (Figure 3E). For the control and continuous groups, the expression was significantly higher at week 2 versus the first two timepoints. The *gsta* expression in the pulse group was also significantly elevated at weeks 2 and 4 versus week 0. For weeks 0 and 2, the expression of *gsta* was significantly lower in the pulse group than the control. The expression of *cu/znsod* and *cat* was not significantly affected by time or treatments (Figures 3B, F).

### 3.4 Histological Status of Mucosal Organs

#### 3.4.1 Gills

**FIGURE 4:**
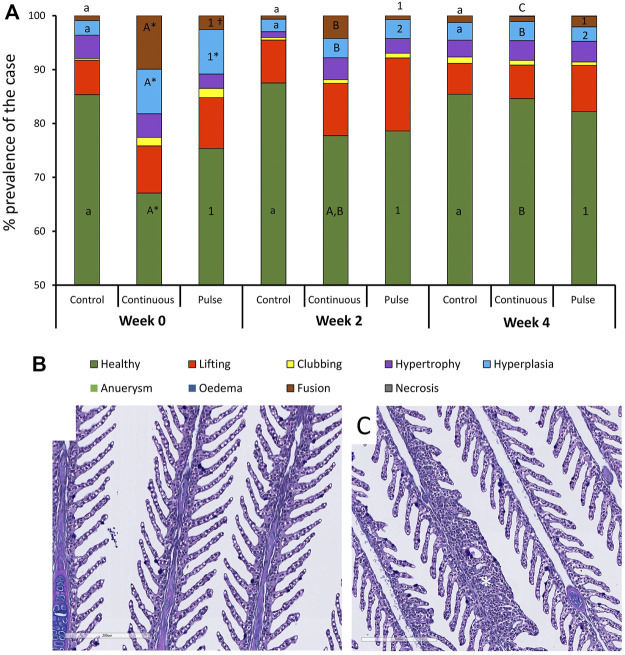
Changes in the structures of the gills following PAA administration. **(A)** Histopathological cases are shown as a percentage of prevalence of a specific lesion to the total analysed cases. Statistical changes over time are indicated by different lower-case letters (for the control group), upper case letters (for the continuous group), or numbers (for the pulse group). Significant differences between the two PAA treatment groups are indicated with a cross (†), while the asterisk (*) represents statistical differences between the treatment group and control. **(B)** A representative section of healthy gills from a control fish where branchial structures are defined; **(C)** A representative section of gills with multifocal hyperplasia where lamellar fusion was also observed (*).

At least 75% of the lamella evaluated here were considered healthy (Figure 4A) except for the continuous group at week 0 that was around 68% and significantly lower than the control group at that timepoint. The number of healthy lamellae in the continuous group increased following PAA application, and the prevalence was above 75% in weeks 2 and 4. Hyperplasia and lamellar fusion were the two lesions identified in the gills of the continuous and pulse groups to be significantly higher than the control at the beginning of the trial (Figures 4B, C). However, obvious recovery was observed at weeks 2 and 4 where the prevalence of these lesions in the continuous and pulse groups was significantly lower than the recorded cases at week 0; neither PAA-exposed group was statistically different from the control group.

### 3.4.2 Skin

**FIGURE 5:**
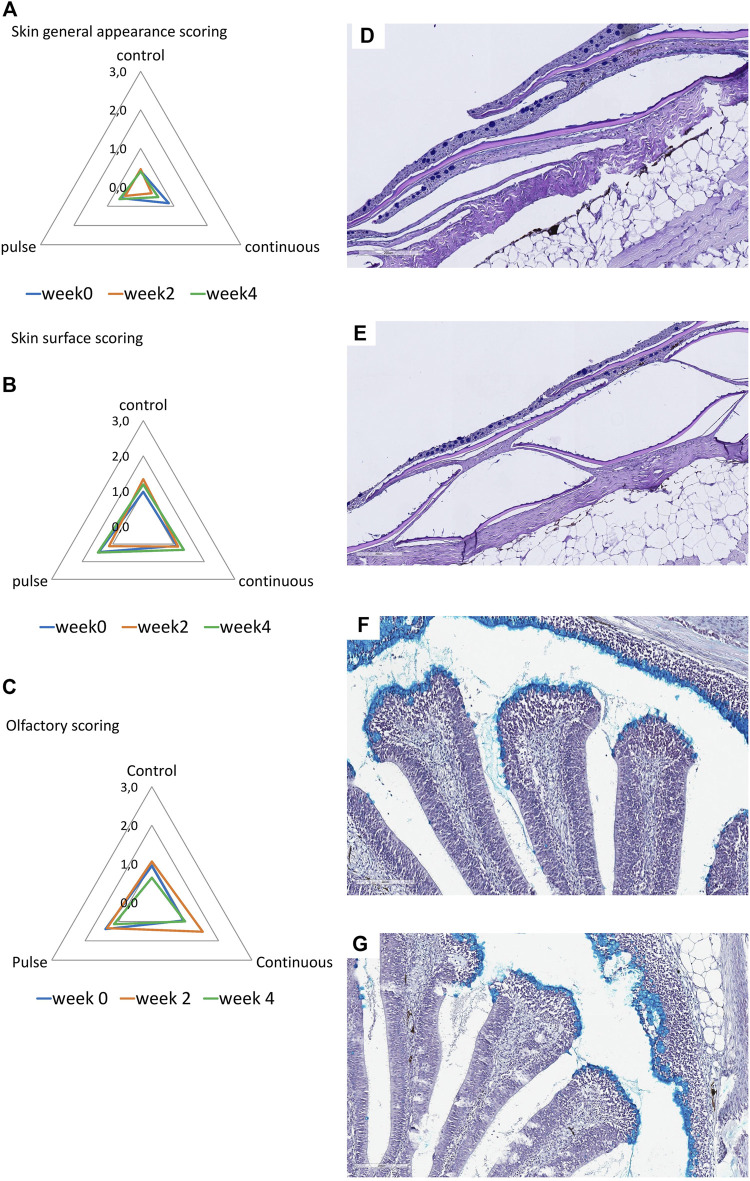
Structural evaluation of the **(A,B)** skin and **(C)** olfactory organ. **(A–C)**: Radar diagrams showing the average scores of qualitative morphological analyses using a scoring scheme (0–3). The skin quality was evaluated by its **(A)** general appearance and **(B)** skin surface quality. **(D,E)**: Representative section of a **(D)** healthy and **(E)** slightly compromised skin. Healthy skin surface is typified by smooth epithelial surface and even presence of epidermis. Roughness and sporadic loss of epidermis are common features of skin with compromised integrity. **(F,G)**: Representative section of the olfactory organ from **(F)** control and **(E)** PAA exposed fish. **(F)** Olfactory lamella of the control fish has well defined structures where mucous cells are densely aggregating at the tip of the mucosa. Mucous cells also line the side of the olfactory mucosal cavity. **(G)** Olfactory lamella missed the structural integrity and include multifocal degeneration of the epithelium though cases such as this are rare.

The general appearance and skin surface quality did not significantly change over time or within treatments (Figures 5A, B). Infrequent cases of scores 1-2 were considered in the continuous group especially in terms of the overall general appearance; in general, the scores in both criteria were predominantly between 0 and 1 in all treatment groups.

### 3.4.3 Olfactory Organ

Histological scores revealed that olfactory organs were minimally affected by the treatments and over time (Figure 5C); nonetheless, scores of 1 to 2 were observed in the continuous group at week 2. No significant inter-treatments were identified.

Morphological measurements of key anatomical structures of the olfactory lamella, i.e., the mucosal tip, lamellar epithelium, and lamina propria, showed temporal differences but were unaffected by PAA exposure. The mucosal tip and lamina propia increased in width and thickness, respectively, in all treatment groups including the control (Table 2).

**TABLE 2:** Morphometries of some key features of the olfactory lamella.

	Week 0			Week 2			Week 4		
	Control	Continuous	Pulse	Control	Continuous	Pulse	Control	Continuous	Pulse
Mucosal tip (µm)	293.1 ± 88.1^a^	280.0 ± 13.3^A^	323 ± 17.9^1^	356.8 ± 21.8^a^	358.1 ± 29.1^B^	324.7 ± 39.3^1^	357.3 ± 57.2^a^	378.7 ± 41.6^B^	361.2 ± 21.9^1^
Olfactory epithelium (µm)	69.6 ± 8.7	67.1 ± 4.4	68.6 ± 6.6	70.6 ± 1.8	71.8 ± 6.3	71.5 ± 5.0	71.2 ± 2.6	70.3 ± 8.6	73.3 ± 1.4
Lamina propria (µm)	57.6 ± 7.8^a^	59.7 ± 6.7^A^	63.1± 11.7^1^	70.9 ± 5.7^b^	71.7 ± 5.2^B^	65.0 ± 4.2^1^	72.4 ± 9.6^b^	74.2 ± 14.2^B^	75.1 ± 7.1^1^

Significant differences over time are represented by different lower-case letters (for control group), upper case letters (for continuous group), or numbers (for pulse group). No significant inter-treatment differences were observed. Values are presented as mean ± SD, of nine individual fish per sampling point.

### 3.5 Mucous Cells in Gills and Skin

The number of mucous cells in the gill filament remained unchanged following PAA treatments (Table 3). The number of mucous cells in the gill lamella in the continuous group was significantly higher at week 4 versus the 2 other time points although no significant inter-treatment differences were observed (Table 3).

**TABLE 3:** Number of mucous cells in the gills and skin.

	Week 0			Week 2			Week 4		
	Control	Continuous	Pulse	Control	Continuous	Pulse	Control	Continuous	Pulse
Gill filament	21.7 ± 11.2	23.7 ± 9.1	25.2 ± 8.5	14.4 ± 6.1	24.6 ± 16.1	22.0 ± 9.5	17.5 ± 4.9	17.4 ± 7.6	16.2 ± 6.9
Gill lamella	19.6 ± 6.8^a^	22.3 ± 10.7^A^	20.7 ± 2.5^1^	19.0 ± 14.0^a^	23.1 ± 8.6^A^	23.4 ± 11.8^1^	27.6 ± 12.9^a^	44.9 ± 17.5^B^	29.1 ± 12.2^1^
Skin	74.0 ± 13.1^a^	64.1 ± 24.4^A^	77.2 ± 18.8^1,2^	81.8 ± 24.2^a^	92.6 ± 30.7^A,B^	107.1 ± 42.9^1^	81.9 ± 33.4^a^	106.1 ± 38.4^B^	74.7 ± 26.7^2*^

Significant differences over time are represented by different lower-case letters (for control group), upper case letters (for continuous group), or numbers (for pulse group). Asterisk (*) indicates that the PAA, treatment group is significantly different from the other PAA treatment group. Values are presented as mean ± SD, of nine individual fish per sampling point.

The mucous cell counts in the skin of the continuous group at week 4 were significantly higher than at the beginning of the trial but not at week 2 (Table 3). For the pulse group, the number of mucous cells at week 4 was significantly lower than at week 2 but was not significantly different from week 0. The number of skin mucous cells in the continuous group was significantly higher than the pulse group at week 4, although neither group had a significant difference versus the control (Table 3).

**FIGURE 6:**
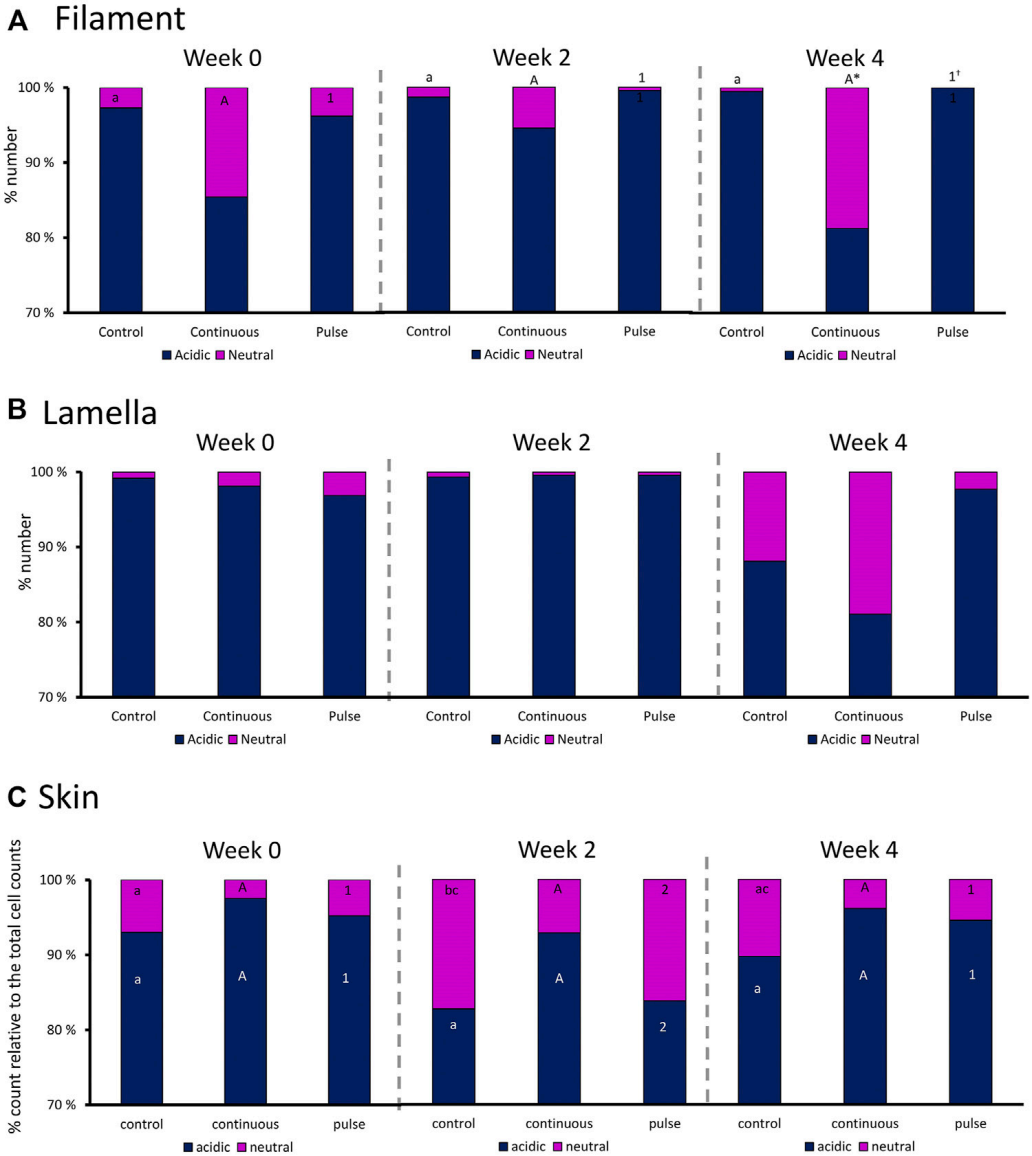
Percentage distribution of acidic (dark blue) and neutral (purple) mucous cells in the gill **(A)** filaments and **(B)** lamella, and in the **(C)** skin. Values are presented related to the total cells counted. Statistical differences over times are indicated by different lower-case letters (for the control group), upper case letters (for the continuous group), or numbers (for the pulse group). The asterisk (*) represents a statistical difference between the treatment group and the control.

We then evaluated the distribution of neutral and acidic mucous in the gills (filament and lamella) and skin (Figure 6). The ratio of acidic and neutral mucous cells in the filament did not significantly vary over time in any groups (Figure 6A). At week 4, the number of neutral mucous cells in the continuous group was significantly higher than the control and the pulse group. The distribution of acidic and neutral mucous cells in the lamella was not affected by time or PAA treatment (Figure 6B).

There were significant temporal differences in the distribution of neutral and acidic mucous cells in the skin of fish from the control and pulse groups (Figure 6C). The number of acidic mucous cells were lower at week 2 in the pulse group, and the number of neutral mucous cells were higher at week 2 than at the two other timepoints. The number of neutral mucous cells in the control group was higher at week 2 than at week 0 but not at week 4.

### 3.6 Total Antioxidant Capacity in Plasma and Skin Mucus at Week 4

**FIGURE 7:**
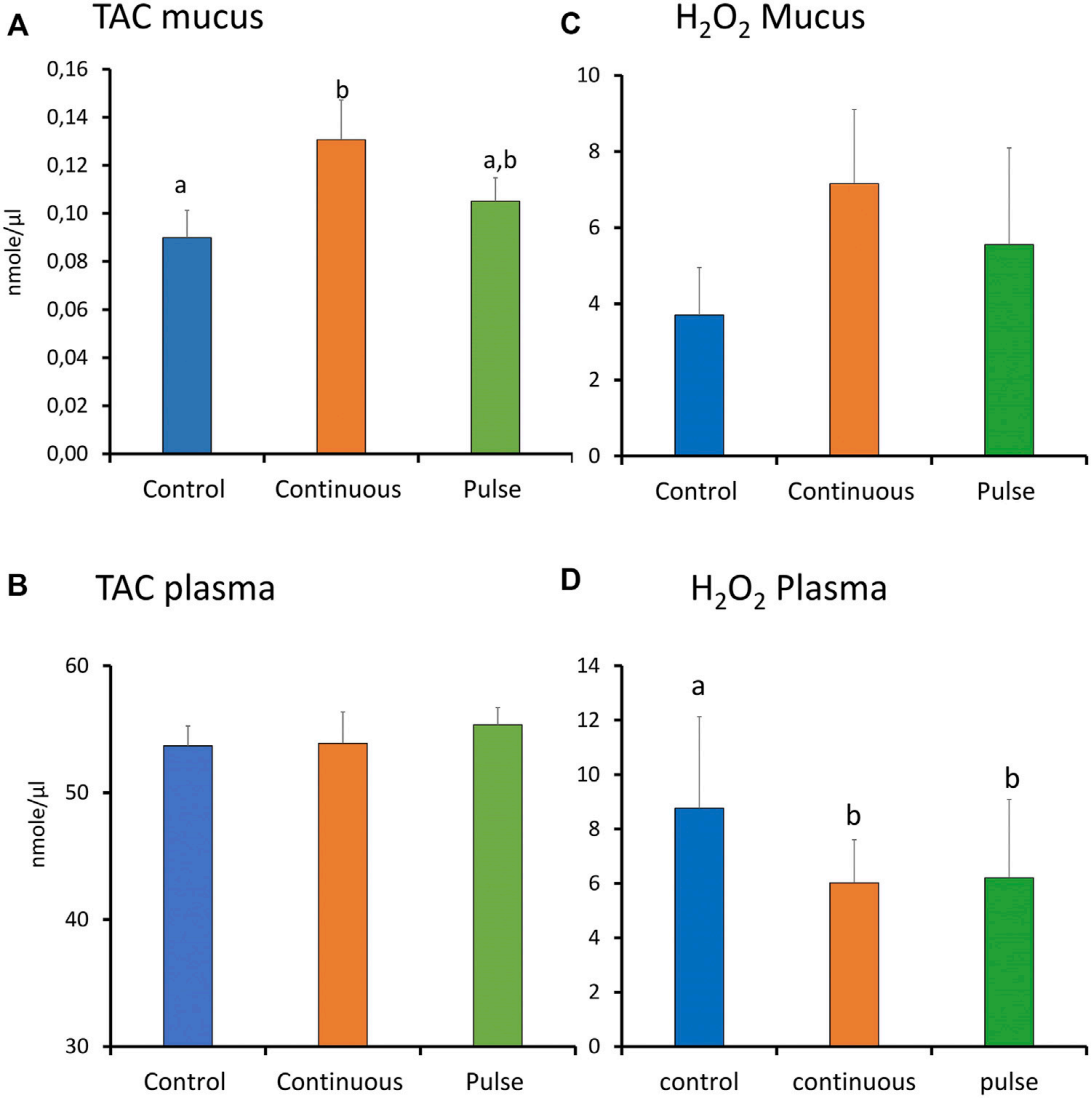
The level of total antioxidants (TAC) and reactive oxygen species (ROS, expressed as H_2_O_2_) in plasma and skin mucus at week 4. Different letters represent statistically significant difference between treatment groups. Values are presented as mean ± SD of nine individual fish.

There were significant inter-treatment differences in the level of TAC in the skin mucus where the level in the continuous group was significantly higher than the control but not the pulse group (Figure 7A). The level of TAC in the pulse group was not significantly different than the control group. Plasmatic TAC level did not show significant inter-treatment differences (Figure 7C).

### 3.7 Reactive Oxygen and Nitrogen Species Level in the Plasma and Skin Mucus at Week 4

The level of ROS (arbitrarily measured as H_2_O_2_) showed no significant differences amongst the treatment groups in the skin mucus (Figure 7B). In the plasma, both continuous and pulse groups showed significantly lower levels of ROS than the control group. No significant difference was observed between the continuous and pulse groups (Figure 7D).

### 3.8 Concentrations of 8OHDG and DIY in Skin, Gills, Dorsal Fin, and Liver Tissue

**FIGURE 8:**
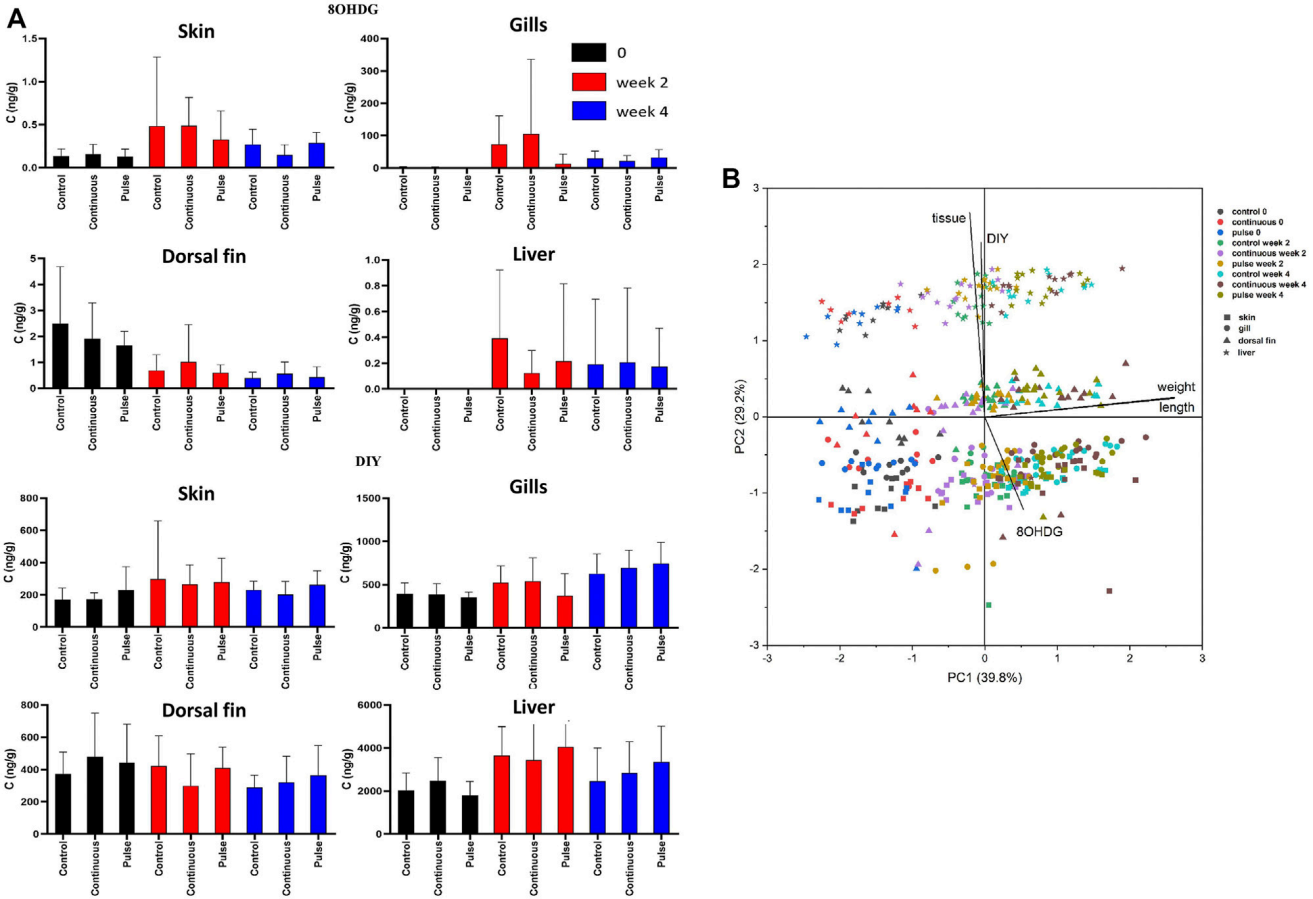
The level of 8OHDG and DIY in Atlantic salmon parr exposed to PAA. **(A)** The levels were quantified in skin, gill, dorsal fin, and liver of experimental fish. Values were presented as mean ± SD of at least 13 fish per treatment group at a particular sampling point. No significant difference was observed over time within the treatment groups. **(B)** Principal component analysis of 8OHDG and DIY concentrations in different tissues of Atlantic salmon parr under different treatments (control, continuous, and pulse) and sampling time points (weeks 0, 2, and 4).

The concentrations of 8OHDG and DIY are presented in Table 4; the concentration distributions are also provided in Supplementary Figure S1. The detection rate% (DR%) of 8OHDG in skin samples was 61%, and the concentrations ranged from <0.11 to 3.60 ng/g wet weight (w.w.) (median: 0.29 ng/g w. w.). DIY was found in 98% of the skin samples at a concentration range of <1.37–1,518 ng/g w. w. (median: 216 ng/g w. w.). The concentration of 8OHDG in fish gill samples ranged from <0.11 to 914 ng/g w. w. (median: 36 ng/g w. w.); DIY was found in 97% of the gill samples ranging from <1.37 to 1,278 ng/g w. w. (median: 534 ng/g w. w). Among the fish dorsal fin samples, 8OHDG was detected with a DR% of 84% with a concentration of <0.11–6 ng/g w. w. (median: 0.55 ng/g w. w.), and the detection rate of DIY was 94% with a concentration range of <1.37–821 ng/g w. w. (median: 373 ng/g w. w.). Among the liver samples, 8OHDG was detected with a DR% of 12% with concentrations ranging from <0.11 to 2.3 ng/g w. w. (median: <0.11 ng/g w. w.). DIY was determined in most samples with a DR% of 95% and a concentration range of <1.37–7,243 ng/g w. w. (median: 2,836 ng/g w. w.). The levels of these markers have differential correlation between tissues such as a positive correlation of DIY level in the gills and liver. There was a negative correlation between the dorsal fin and the gills (Supplementary Tables 2, 3). The distribution of the 8OHDG and DIY concentrations in the four different tissues with different treatments (control, continuous, pulse) and sampling times (week 0, 2 and 4) are presented in Figure 8A and visualised through PCA in Figure 8B. Though apparent temporal variability was observed, these changes were not statistically significant. Moreover, no inter-treatment differences were observed.

**TABLE 4:** Concentration (ng/g wet weight) of 8OHDG and DIY in skin, gill, dorsal fin, and liver of Atlantic salmon parr.

			Week 0			Week 2			Week 4		
			Control	Continuous	Pulse	Control	Continuous	Pulse	Control	Continuous	Pulse
**8OHDG**	skin	min	<0.11	<0.11	<0.11	<0.11	<0.11	<0.11	<0.11	<0.11	<0.11
		max	0.26	0.35	0.26	3.1	1.1	0.93	0.65	0.35	0.52
		median	0.13	0.14	<0.11	0.2	0.61	0.22	0.26	<0.11	0.28
		mean	0.13	0.16	0.13	0.48	0.49	0.33	0.27	0.15	0.29
	gill	min	1.2	<0.11	0.73	<0.11	<0.11	0.06	1.5	8.1	8.1
		max	6.5	4.1	1.8	232	914	118	81	71	90
		median	1.8	2.1	1.4	45	7.2	2	26	17	24
		mean	2.6	1.9	1.3	73	105	13	29	22	33
	dorsal fin	min	<0.11	<0.11	1	<0.11	<0.11	<0.11	<0.11	<0.11	<0.11
		max	5.7	4.1	2.5	2.3	6	1.2	0.9	1.6	1.3
		median	2.6	1.9	1.4	0.64	0.61	0.5	0.35	0.51	0.36
		mean	2.5	1.9	1.7	0.69	1	0.6	0.4	0.57	0.43
	Liver	min	<0.11	<0.11	<0.11	<0.11	<0.11	<0.11	<0.11	<0.11	<0.11
		max	<0.11	<0.11	<0.11	2	0.59	2.3	1.9	2.3	0.96
		median	<0.11	<0.11	<0.11	<0.11	<0.11	<0.11	<0.11	<0.11	<0.11
		mean	<0.11	<0.11	<0.11	0.39	0.12	0.22	0.19	0.21	0.17
**DIY**	skin	min	97	119	133	<1.4	82	114	125	<1.4	153
		max	275	232	592	1,518	446	540	346	321	487
		median	132	163	168	175	239	230	229	205	252
		mean	172	172	230	299	266	279	229	201	262
	gill	min	300	182	282	221	224	<1.4	67	418	266
		max	718	654	481	888	1,234	943	966	1,278	1,165
		median	364	369	333	564	585	368	568	663	700
		mean	394	384	352	522	542	371	628	695	744
	dorsal fin	min	210	<1.4	<1.4	20	<1.4	208	182	<1.4	<1.4
		max	533	821	777	791	541	679	413	548	747
		median	395	601	522	425	366	431	277	348	348
		mean	372	479	444	421	299	409	288	319	365
	liver	min	786	894	611	1728	<1.4	2,308	<1.4	<1.4	<1.4
		max	3,357	4,269	2,533	6,404	7,243	7,074	5,365	4,969	5,582
		median	2030	2,452	2015	3,450	3,137	3,927	2,145	3,335	3,343
		mean	2019	2,478	1795	3,647	3,435	4,040	2,464	2,834	3,349

N = 115 (skin); 117 (gills); 114 (dorsal fin); 113 (liver).

## 4 Discussion

PAA is a disinfectant with several advantages for improving the rearing environment, but there are still risks involved in its use (Pedersen et al., 2009; Pedersen et al., 2013; Pedersen and Lazado, 2020; Dicocco et al., 2021). Therefore, exploring its application as a disinfectant in RAS must be supported by data on its health impacts on fish. Current evidence on the health effects on salmon are seen during the smolt stage (Lazado et al., 2020a; Lazado et al., 2021a; Lazado et al., 2021b; Osório et al., 2022). Existing work often assumes that PAA will have the same impacts on parr, but such assumptions are tenuous due to immense physiological differences between parr and smolts. We recently reported an acute dose-response of salmon parr to PAA; the no-observed-effect concentration was below 1.6 mg/L. Concentrations higher than 3.2 ml/L posed severe health risks to parr (Mota et al., 2022a). This new insight further highlighted that responses of salmon parr and smolts differ substantially; earlier results demonstrated that smolts could even tolerate an acute exposure as high as 4.8 mg/L (Lazado et al., 2021b). Here, we chronically exposed parr to 1 mg/L PAA and documented physiological and structural consequences if the oxidant was administered either in pulse or continuous modes. The general outcome suggests that the duration of the trial and not the mode of PAA administration had a more significant impact on the physiological and structural changes.

### Modulation of Antioxidant Gene Expression Following PAA Exposure Differs Among Mucosal Organs

One of the main consequences of PAA administration identified previously in salmonids was the induction of oxidative stress that could be both transient and persistent depending on the dose, frequency of exposure, and influence of a confounding stressor (Soleng et al., 2019; Liu et al., 2020; Lazado et al., 2021b). PAA degrades into free radicals upon contact with water, and these active molecules provide the major disinfection power of PAA and simultaneously present a potential stressor for the fish.

Mucosal surfaces such as the skin, gills, and the olfactory organ are at the interface of the internal and external environments; thus, they are often vulnerable to chemotherapeutics-induced oxidative stress. Hence, several players in immune defence in these mucosal surfaces are expected to mount responses to counteract the physiological pressures from environmental stressors so as to not further trigger systemic dysregulation. The modulation of antioxidant defence genes in the gills, skin, and olfactory organs in this study) demonstrated that PAA could be a potential environmental stressor (Figures 1–3, however, the impact collectively suggests that the risk was minimal at least in terms of the biomarkers analysed here. This corroborates a recent study of salmon smolts where PAA addition in brackish water RAS was identified as a mild environmental stressor that triggered local and systemic oxidative stress (Osório et al., 2022). In earlier studies, the gills of salmon smolts were the most responsive to PAA-induced oxidative stress although we could not establish such a striking profile here.

The manganese superoxide dismutase (*mnsod*) is an important mitochondrial antioxidant enzyme that detoxifies the free radical superoxide—the major byproduct of mitochondrial respiration (Candas and Li, 2014). There was a transient upregulation of *mnsod* in the skin of the pulse group at week 2; its expression was not affected in the olfactory organ or the gills, thus suggesting that detoxification through *mnsod* was more active in the skin than in other mucosal organs (Figures 1–3). The role of *mnsod* in oxidative stress in salmon is often studied in the gills and liver (Olsvik et al., 2011; Solberg et al., 2012; Soleng et al., 2019); thus, these results offer insight into the role of this molecule to cutaneous response to an oxidative stressor.

Glutathione reductase (*gr*) plays an essential role in ROS cellular scavenging. Glutathione is maintained in its reduced form and can detoxify xenobiotics (Srikanth et al., 2013). The upregulation of this gene in both the gills and skin of PAA-exposed fish at week 4 indicates its involvement in the mucosal detoxification of PAA (Figures 1, 2). *Gr* had earlier been implicated as an essential molecule in the recovery of the gills from a single PAA exposure in salmon smolts (Soleng et al., 2019). We found that *gr* was upregulated in the skin of smolts following intermittent administration of PAA (Osório et al., 2022). Our data underscore the crucial role of *gr* in the adaptive responses to PAA in salmon; this is a potential marker for PAA-induced oxidative stress response and is perhaps not dependent on life stage.

Glutathione peroxidase (*gpx*) is an enzyme that protects fish from the damage caused by H_2_O_2_; GPX reduces H_2_O_2_ to lipid hydroperoxides (Srikanth et al., 2013). Increased endogenous levels of *gpx* could be an important strategy to resolve oxidative stress-induced pathologies (Prasad et al., 2018). Pulse delivery of PAA had a significant impact on *gpx* expression in the skin (Figure 2). Such PAA-induced modulation in the skin had not been observed in earlier studies of salmon smolts (Soleng et al., 2019; Osório et al., 2022). Our results suggest that the antioxidant function of *gpx* against PAA is likely influenced by the life stage of salmon; it might be crucial during the parr stage. Glutathione S-transferases (*gsta*) are a superfamily of multifunctional detoxification isoenzymes and play an important role in cellular signalling (Özaslan et al., 2017). Transcription was affected in both the olfactory organ and the gills of PAA-exposed groups. Moreover, two different profiles were demonstrated: upregulation in the gills and downregulation in the olfactory organs—particularly in the pulse group (Figures 1, 3). These data suggest that *gsta*-mediated detoxification of PAA perhaps followed distinct kinetics of activation. The time when its expression was affected by PAA (week 2 in the olfactory organ versus week 4 in the gills) partially support this implication.

The inter-treatment differences in the expression of *gr*, *gpx*, and *gsta* in the olfactory organ of the pulse group at week 0 was quite a surprising result and challenging to explain. We checked the water quality parameters and no indications of deviations in this group at the start of the trial. Moreover, the histological section of the olfactory organ showed no apparent differences among the groups at the beginning. It could be possible that regulation of these oxidative stress genes may be related to handling stress during sampling, and we hope to provide more insights into this interplay in the future. We are currently exploring the sensitivity of the olfactory organ to handling and crowding stress.

The expression of the selected antioxidant biomarkers was affected by time (Figures 1–3), which indicates that they have a basal physiological role to maintain the internal redox homeostasis concerning growth/ageing in addition to counteracting exogenous radicals (Valko et al., 2007; Tan et al., 2018). Overall, the physiological changes reported here indicate that growth may strongly contribute to the expression of antioxidant defences in mucosal tissues of salmon parr; the oxidant treatments applied here only provided a minimal influence. This observation is beneficial because it indicates that the treatments did not trigger a considerable dysregulation of mucosal oxidative stress status.

### Imbalance in ROS and TAC Levels Is Found in Skin Mucus and Plasma

PAA produces radicals during its decay, and they are triggers of the oxidative response of the host (Liu et al., 2020; Pedersen and Lazado, 2020). This interaction may also result in an internal imbalance, and exogenous production of reactive oxygen species (ROS) by the host. To study this interaction in the mucosa, we used the skin mucus to analyse total antioxidant capacity (TAC) and the levels of ROS (Figure 7). Due to the small size of the fish, we only managed to collect a sufficient amount of skin mucus for analysis at week 4. There was a significant increase in TAC in the skin mucus of fish from the continuous group, thus indicating that the treatment triggered a humoral antioxidant response, although this did not agree with the gene expression data. Though no significant inter-treatment differences were observed in terms of ROS level in the skin mucus, the trend indicates elevated levels in the continuous group as well. The increase in TAC and ROS in the continuous group suggests that the humoral antioxidants maintained the balance of ROS.

Earlier studies of salmon smolts showed that PAA could trigger oxidative stress at the systemic level (Soleng et al., 2019; Lazado et al., 2020a; Osório et al., 2022). This was documented either by an increase in plasmatic TAC or regulation of metabolites with known function in the antioxidant-oxidant homeostasis. Here, we found that systemic TAC was not significantly altered. However, we note the significantly lower level of plasmatic ROS in both groups. These lower levels imply that the exposure to PAA, regardless of administration mode, did not promote exogenous production of ROS. This did not agree with previous studies on salmon smolts. The PAA-induced ROS production of smolts is likely more sensitive in parr and could be attributed to their physiological differences. Alternative, the systemic oxidative stress caused by PAA might be dose-dependent. The latter has been observed earlier in smolts (Soleng et al., 2019).

### Markers of Oxidative Stress-Induced Damage of DNA and Proteins in the Gills, Skin, Dorsal Fin, and Liver Tissue Are Not Affected by PAA

We further studied additional markers of oxidative stress using a newly developed method of simultaneously detection of 8-OHDG and DIY using UPLC^®^–ESI (electrospray)–MS/MS analysis (Zhang et al., 2022). DNA and protein damage is often the product of the inability of the organism to counteract oxidative stress. During this process, 8-OHDG and DIY are formed and are thus considered specific biomarkers of DNA and protein damage from oxidative stress (Malencik and Anderson, 2003; Valavanidis et al., 2009). There were no significant differences in the concentration profiles of 8OHDG and DIY across the three treatment groups (continuous, control, pulse), thus indicating that 1 mg/L PAA had minimal damaging effects on proteins, and DNA that could have caused an increase in oxidative stress markers 8OHDG and DIY in the four main organs of the fish (Figure 8). Pearson correlation analysis indicated that the concentration of DIY in liver and gills were significantly positively correlated, while the concentration of 8OHDG in the dorsal fin was negatively correlated with that in skin. The positive correlation for DIY indicates common oxidative effects in the proteins of the liver and gills. In contrast, 8OHDG had a negative correlation for DNA oxidation between the dorsal fin and skin. It is well documented that the gills and olfactory organ of salmon have more sensitive antioxidant systems than the skin to PAA (Soleng et al., 2019; Lazado et al., 2020a; Osório et al., 2022), indicating that different tissues can respond differently to oxidative stress. The opposite effects that we observed in the oxidation of DNA between the dorsal fin and skin can also be potentially attributed to such differences in their antioxidant systems.

### Phenotypic Responses to PAA Are Revealed by the Structural and Morphometric Changes in the Mucosal Organs

The gills of salmon parr were responsive to PAA (Figure 4) and corroborate the results from previous PAA trials in smolts; the gills were affected at varying magnitudes by PAA even at nominal concentrations and short-lived exposure (Lazado et al., 2021b; Haddeland et al., 2021). In addition, the responses seen here agreed with recently published data on a dose-response study on PAA in salmon parr (Mota et al., 2022a). The alterations found here were minimal, and lesions were considered non-specific responses to a RAS environment (Good et al., 2011; Lazado et al., 2021a). There were significant inter-treatment differences in the number of healthy lamellae at week 0. The two groups that were eventually exposed to PAA and showed a lower count than the control. We could not associate this difference with PAA because treatments had not yet started when these samples were taken; hence, this difference could be stochastic because there were no significant inter-treatment differences following treatment. The lesions (i.e., fusion and hyperplasia) found at week 0 in the continuous and pulse groups did not increase following PAA treatments. This result signifies that PAA administration, regardless of the mode of delivery, could provide a favourable environment for potential recovery of the gills. This also further indicates that salmon gills could adapt to the presence of low-level oxidants in the water; thus supporting the well-documented remodelling capacity of fish gills.

The olfactory organ of fish is where olfaction and immunity converge, and thus waterborne chemical hazards could influence its structural features. PAA is a potential respiratory tract irritant in humans (Hawley et al., 2017). Earlier evidence in salmon smolt indicate that PAA is a chemical stressor to the olfactory organ (Lazado et al., 2020b; Osório et al., 2022). Here, we report that PAA could affect the structural integrity of the olfactory organ especially at week 2 in the continuous group (Figure 5). However, the overall impact is slight because tissue health scores were less than 2, which is arbitrarily considered moderate. The mucosal cells in smolts were tightly and densely distributed on the surface of the olfactory lamella of salmon. This feature could have protected the olfactory mucosa from potential irritation from PAA.

Of all the mucosal organs, the skin was least impacted by PAA (Figure 5). The overall appearance of the skin and its quality of the epithelium were not significantly affected by PAA. The structural organisation of the skin consisted of several layers, which made it less susceptible to PAA than the gills and the olfactory organ. This response profile of salmon skin had earlier been documented in salmon exposed to PAA (Lazado et al., 2020a; Mota et al., 2022a; Osório et al., 2022).

Mucus-secreting cells (i.e., goblet cells, sacciform cells, and club cells) are important cellular components of the fish mucosa (Shephard, 1994; Pittman et al., 2011). These mucosal sentinels respond to environmental factors by changing the chemical and physical properties of the mucus and/or induce hyperplasic/hypertrophic phenotypic changes. This work showed that PAA administration did not significantly affect the number of mucous cells in the gills and skin of salmon parr regardless of the mode of delivery (Figure 6; Table 2). The mucus cells in the gills and skin of salmon parr are likely less susceptible to PAA unlike in smolts where phenotypic changes in mucous cells characterise the striking response to PAA (Haddeland et al., 2021). Though the skin structures were less affected by PAA, the distribution of neutral and acidic mucous cells was impacted in the pulse group. These dynamic changes indicate metabolic adaptations of mucous cells to PAA, thus ensuring the barrier defence in the skin mucosa.

## Conclusion

This study illustrates that low-dose PAA (1 mg/L) does not pose a significant threat to salmon parr health in a freshwater RAS. The changes observed here for the molecular and phenotypic indicators collectively reveal that PAA could trigger stress—especially at the mucosa; however, this consequence was not chronic. The alterations observed here were within the expected physiological responses of the organism, and thus can be considered compensatory to the demands of the external stress stimulus. The response profiles of the experimental fish suggest that the mode of delivery had little impact on the responses to PAA. The results expand the community’s understanding of the biological aspects of PAA use in aquaculture and support the use of PAA as a routine water disinfection for salmon RAS. This study will facilitate opportunities to optimise the application of PAA in RAS.

## Data Availability

The original contributions presented in the study are included in the article/Supplementary Material, further inquiries can be directed to the corresponding author.
