# Supplementary material for: Mode of Application of Peracetic Acid-Based Disinfectants has a Minimal Influence on the Antioxidant Defences and Mucosal Structures of Atlantic Salmon (Salmo salar) Parr
Source: Front Physiol. 2022 May 25;13:900593. doi: 10.3389/fphys.2022.900593 (PMC9174794; doi:10.3389/fphys.2022.900593)
Supplement: Supplementary file 1 [file Table1.DOCX]

Supplementary Material

Supplementary Table 1. Scoring model of the olfactory organ.

| **Score** | **Description** |
| --- | --- |
| 0 | Structure is intact and well-defined, epithelial surface is smooth |
| 1 | 10-30% show tissue damage including loss of well-defined structure, epithelial surface shows roughness |
| 2 | >30-60% show tissue damage including loss of well-defined structure, epithelial surface shows roughness |
| 3 | >60 or complete loss of tissue structure including sever signs necrosis/degeneration |

**Supplementary Table 2. Concentrations of 8OHDG and DIY in skin, gill, dorsal fin and liver samples**

| **ng/g** | **Skin**  **(N=115)** | | **Gill**  **(N=117)** | | **Dorsal fin**  **(N=114)** | | **Liver**  **(N=113)** | |
| --- | --- | --- | --- | --- | --- | --- | --- | --- |
|  | 8OHDG | DIY | 8OHDG | DIY | 8OHDG | DIY | 8OHDG | DIY |
| Min | <0.11 | <1.37 | <0.11 | <1.37 | <0.11 | <1.37 | <0.11 | <1.37 |
| max | 3.1 | 1518 | 914 | 1278 | 6 | 821 | 2.3 | 7243 |
| median | 0.22 | 216 | 36 | 534 | 0.55 | 373 | <LOD | 2836 |
| DR | 61 | 98 | 92 | 97 | 84 | 94 | 12 | 95 |

**Supplementary Table 3. Correlation of concentration of 8OHDG and DIY in different fish tissues**

| **DIY** | **skin** | **gill** | **dorsal fin** | **liver** |
| --- | --- | --- | --- | --- |
| skin |  | 0.191 | -0.340 | 0.153 |
| gill |  |  | -0.103 | 0.452^**^ |
| dorsal fin |  |  |  | 0.064 |
| liver |  |  |  |  |
| 8-OHDG | skin | gill | dorsal fin | liver |
| skin |  | 0.045 | -0.061 | -0.688 |
| gill |  |  | -0.230^*^ | -0.198 |
| dorsal fin |  |  |  | 0.024 |
| liver |  |  |  |  |

^*^: *p*<0.05; ^**^: *p*<0.01

**Supplementary Figure 1. Distribution of 8OHDG and DIY in skin, gill, dorsal fin and liver. A-B**) The blue line represents median value, the up and down green line represent 25% and 75%.
